# The Relationship between Food Security Status and Fruit and Vegetable Intake during the COVID-19 Pandemic

**DOI:** 10.3390/nu13030712

**Published:** 2021-02-24

**Authors:** Michelle M. Litton, Alyssa W. Beavers

**Affiliations:** Department of Nutrition and Food Science, Wayne State University, Science Hall 410 W Warren, Detroit, MI 48201, USA; gs6226@wayne.edu

**Keywords:** COVID-19, food insecurity, fruit, vegetable

## Abstract

The coronavirus disease 2019 (COVID-19) pandemic has drastically altered food shopping behaviors, and the resulting economic recession has caused a spike in food insecurity. Since food insecurity is associated with poor diet, especially low intake of fruits and vegetables, food-insecure individuals may disproportionately experience negative health impacts related to poor diet during the pandemic. To assess the relationship between food security status and fruit and vegetable intake during the COVID-19 pandemic, we conducted an online survey of adult residents of the US state of Michigan in June of 2020. Among the 484 survey respondents, 36.2% were classified as food-insecure. Food-insecure respondents consumed fruits and vegetables fewer times per day than food-secure respondents and were more likely to report decreasing their consumption of any type of fruits and vegetables (total, fresh, frozen, and canned) since the pandemic started. For those who reduced their purchase of fresh fruit and vegetable, reasons included poor quality, poor availability, high price, reduced store trips, and concerns of contamination. These findings highlight the need for adequate food assistance during the COVID-19 pandemic and in future pandemics, as well as public health messages that promote healthy eating.

## 1. Introduction

The coronavirus disease 2019 (COVID-19) pandemic has resulted in a global recession, leading to a drastic increase in unemployment and a concomitant spike in food insecurity worldwide [1,2,3,4]. Food insecurity is characterized by “reduced quality, variety, or desirability of diet” and is accompanied by reduced intake of food quantity in severe forms of food insecurity [5]. Food insecurity is deeply linked to national and household-level economic conditions. In the United States, the rise in unemployment caused by stay-at-home orders has been a major contributor to the rise in food insecurity since the start of the COVID-19 pandemic [6]. It is estimated that the United States food insecurity rate doubled in the wake of COVID-19, reaching 22.8% in April of 2020 [7]. By April 2020, the U.S. Bureau of Labor Statistics reported that the national U.S. unemployment rate reached 14.7%, an all-time high since the Great Depression of 1933 [8,9]. The U.S. Congressional Budget Office forecasts that unemployment may not return to pre-pandemic levels until after 2030, indicating that the food insecurity consequences of COVID-19 will be long-lasting [10]. 

Food insecurity may have negative health impacts due to its association with poor diet quality. Several studies have found that Healthy Eating Index scores are lower among low-income or food-insecure individuals, [11,12,13], and food insecurity is associated with lower fruit and vegetable intake [12,14]. The intake of fruits and vegetables is associated with reduced risk of chronic diseases, including type 2 diabetes and cardiovascular disease, as well as with lower mortality [15,16,17,18]. Thus, low fruit and vegetable intake may in part contribute to the increased prevalence of chronic diseases, such as diabetes and hypertension, that is associated with food insecurity [19,20]. The link between food insecurity, diet quality, and chronic diseases is of particular concern during the COVID-19 pandemic. People with chronic diseases, such as kidney disease, diabetes, hypertension, and cardiovascular diseases, are at an increased risk for severe COVID-19 infection and mortality [21,22]. Since food insecurity is associated with chronic diseases, it is possible that food-insecure individuals have experienced a disproportionate burden of the negative health impacts and mortality from COVID-19. 

Since the immune system is influenced by nutrition, eating a healthy diet might reduce the risk of negative outcomes from COVID-19 infection [23,24]. A diet rich in fruits and vegetables may be especially important, as these foods contain high levels of anti-inflammatory compounds such as carotenoids, vitamin C, and phytochemicals [23,24]. Unfortunately, eating a healthy diet has become more challenging during the COVID-19 pandemic. Those who have become unemployed may have less money to spend on nutritious food, and other pandemic-related factors have influenced food availability and food-purchasing habits. Disruptions in the food supply chain and “panic buying” led to reduced availability of some foods [25,26]. Several studies have demonstrated that reduced frequency of grocery shopping in order to reduce exposure to the virus [27,28] has resulted in decreased purchase of perishable foods such as fresh fruits and vegetables [29]. However, existing research has not examined the relationship between food security status and fruit and vegetable purchase and consumption during the COVID-19 pandemic. This knowledge is important for understanding disparities in dietary habits that may put food-insecure individuals at higher risk of short- and long-term poor health outcomes. This research can also aide in identifying strategies to improve dietary intake. This study aimed to examine change in purchase and consumption of fruits and vegetables during the COVID-19 pandemic and determine if this differed by food security status. Additionally, this study aimed to examine perceived reasons for change in fruit and vegetable purchases during the COVID-19 pandemic.

## 2. Materials and Methods

An online survey was conducted via Qualtrics from 17 June to 29 June 2020. The survey was advertised by using paid Facebook advertisements. Participants were eligible for the survey if they lived in the state of Michigan and were at least 18 years of age. Blank survey responses (*n* = 41), surveys completed in less than half the median time (*n* = 82), surveys with out-of-Michigan zip codes (*n* = 2), responses that were not submitted (*n* = 68), responses with inconsistencies (*n* = 25), and responses without food security data (*n* = 11) were excluded. After these exclusions, 484 surveys remained for analysis. This study was approved by the Wayne State University Institutional Review Board.

### 2.1. Independent Variables

Demographic information included age, race and ethnicity, gender, education, and income. Food security over the past 30 days was measured using the United States Department of Agriculture (USDA) six-item short-form household food security module, and participants were classified as either food-secure or food-insecure [30].

### 2.2. Outcomes

To assess change in the amount of fresh fruits and vegetables purchased since the beginning of the pandemic, participants were asked to indicate if they purchased “a lot less,” “a little less,” “the same,” “a little more,” or “a lot more” since the beginning of the COVID-19 pandemic. This question was adapted from the “Food access and food security during COVID-19 Survey Version 2.1” [31]. If participants indicated they were purchasing more or less fresh fruits and vegetables, they were presented with an open-ended question to explain why: “Please explain why you are purchasing (more or less) fresh fruits and vegetables.” 

The National Cancer Institute’s Dietary Screener Questionnaire was used to assess the frequency of fruit and vegetable consumption [32]. Self-reported change in fruit and vegetable intake was assessed by asking participants to indicate if their consumption of total fruits and vegetables, as well as of different forms of fruits and vegetables (canned, frozen, and fresh), had changed in the past month compared with before the COVID-19 outbreak. Response options for these questions were “less,” “the same,” and “more.” These questions were also adapted from the “Food access and food security during COVID-19 Survey Version 2.1” [31].

Additional questions were included to understand factors that may influence purchase or consumption of fruits and vegetables. To assess the quality of produce available in participants’ neighborhoods, participants were asked to rate their agreement with the statement “The fresh produce in my neighborhood is of high quality” from the “Perceived Nutrition Environment Measures Survey” (NEMS-P) [33]. To assess the perceived impact of cost on fruit and vegetable consumption, participants were asked to indicate their agreement with the statement “I don’t eat fruits and vegetables as much as I like to because they cost too much” from the “Food Attitudes and Behaviors” (FAB) survey [34]. 

### 2.3. Analysis

To examine differences in demographic characteristics between food-secure and food-insecure respondents, Wilcoxon rank sum test was used for ordinal variables, and Fisher’s exact test or chi square test was used for categorical variables. Regression analyses were conducted to examine the association between food security status and each outcome variable. Linear regression was used for the outcome variable frequency of fruit and vegetable consumption in times per day. This variable was log-transformed to meet the assumptions of multiple linear regression. All other outcome variables (change in fresh, frozen, canned, and total fruit and vegetable intake; change in fresh fruit and vegetable purchase; perceived neighborhood quality of produce; and cost as a perceived barrier to fresh fruit and vegetable consumption) were on an ordinal scale. For these variables, the gologit2 command in Stata was used to perform separate generalized ordered logistic regression models for each outcome. This approach is appropriate when the proportional odds assumption of ordered logistic regression is violated, as occurred for this data. For each of the eight outcome variables, two separate models were applied. Model one was unadjusted and included only food security status as a predictor. Model two added age and race/ethnicity as control variables. Race and ethnicity were collapsed into a binary variable (whether respondent was or was not non-Hispanic white) due to the low number of respondents who were non-white or Hispanic. The open-ended questions regarding why participants purchased more or less fruits and vegetables since the start of the COVID-19 pandemic were analyzed using qualitative thematic coding. Two coders coded each response independently. Consensus was reached for coding differences and was facilitated by the principal investigator. 

## 3. Results

Demographic characteristics, overall and by food security status, are found in Table 1. The overall prevalence of food insecurity was 36.2%. Gender significantly differed between food-secure and food-insecure respondents, but respondents were overwhelmingly female in both groups, 94.8% and 96.6%, respectively. A large share of respondents, 89.6%, were white, and the average age of the respondents was 46. A significantly higher percentage of food-insecure respondents reported being Hispanic or Latino/a, but the percent was low for both groups (2.3% for food-secure respondents, and 6.3% for food-insecure respondents). A significantly higher percentage of food-insecure households had at least one child (62.1% compared with 42.6%), and a significantly higher percentage of food-insecure households had experienced employment disruption (being furloughed, having reduced hours, or being laid off) since the pandemic started, i.e., 75.5% compared with 53.9%. Income and education were significantly different between food-secure and food-insecure respondents. 

Descriptive statistics for each outcome variable by food security status are found in Table 2. On average, food-secure respondents consumed fruits and vegetables 3.33 times per day, compared with 2.99 times per day for food-insecure respondents. A lower percentage of food-insecure respondents reported consuming the same amount of total, fresh, frozen, and canned fruits and vegetables since the start of the COVID-19 pandemic, indicating that food-insecure respondents have made considerably larger changes to fruit and vegetable consumption since the start of the pandemic. The largest differences between food-secure and food-insecure respondents were in the percentage corresponding to consuming less of each type of fruits and vegetables, indicating that consuming less was approximately two to three times more frequent among food-insecure respondents. A higher percentage of food-insecure respondents reported cost as a barrier to eating fruits and vegetables, and a lower percentage reported that fruits and vegetables in their neighborhood were high-quality.

The log of fruit and vegetable consumption frequency was significantly lower for food-insecure respondents compared with food-secure respondents (for model 1 β = −0.19, *p* = 0.002; for model 2 β = −0.20, *p* = 0.002). The remaining regression models used generalized ordered logistic regression and are found in Table 3. Odds ratios (OR) for these models are interpreted as the odds of any higher response category than the given response. For change in total, fresh, canned, and frozen fruit and vegetable consumption since the pandemic’s start, response options from lowest to highest were less, same, and more. For total fruits and vegetables, food-insecure respondents were significantly less likely than food-secure respondents to report a higher response than consuming less (OR = 0.22, *p* < 0.001) and significantly less likely to report a higher response than consuming the same (OR = 0.56. *p* = 0.003). Findings were similar for fresh vegetables: for consuming less, OR = 0.33 and *p* < 0.001, and for same consumption, OR = 0.53, *p* = 0.003. For frozen vegetables, food-insecure respondents were significantly less likely to report a higher response than consuming less (OR = 0.28, *p* < 0.001). These results indicate that food-insecure respondents were more likely than food-secure respondents to report consuming less fresh, total, and frozen fruits and vegetables than they consumed before the pandemic. For canned fruits and vegetables, food insecure-respondents were significantly less likely to report a higher response than less consumption (OR = 0.22, *p* < 0.001) and were significantly more likely to report a higher response than the same consumption (OR = 1.95, *p* = 0.004). This indicates that food-insecure respondents were more likely than food-secure respondents to report either increasing or decreasing their consumption of canned fruits and vegetables. 

For questions related to cost and quality of fresh produce, responses options from lowest to highest were strongly disagree, somewhat disagree, neither agree or disagree, somewhat agree, and strongly agree. Perceived quality of fresh produce was assessed by participants’ level of agreement with the statement “The fresh produce in my neighborhood is of high quality.” Food-insecure respondents were significantly less likely to report responses higher than neither agree nor disagree (OR = 0.41, *p* < 0.001) and somewhat agree (OR = 0.25, *p* < 0.001). This signifies that food-insecure respondents perceived the produce in their neighborhood to be of lower quality than food-secure respondents did. The perceived influence of cost as a barrier to purchasing fresh produce was assessed with participants’ level of agreement with the statement “I don’t eat fruits and vegetables as much as I like to because they cost too much.” Food-insecure respondents were significantly more likely than food-secure respondents to report any higher response for all response levels, with OR ranging from 3.86 to 7.95, *p* < 0.001 for each. These results indicate that food-insecure respondents are substantially more likely to perceive cost as a barrier to eating fruits and vegetables. 

For the question assessing change in the purchase of fresh fruits and vegetables, response options from lowest to highest were a lot less, a little less, the same, a little more, or a lot more. Food-insecure respondents were significantly less likely than food- secure respondents to report any higher response than a lot less (OR = 0.34, *p* = 0.004), a little less (OR= 0.39, *p* < 0.001), and same (OR = 0.62, *p* = 0.023). Thus, food-insecure respondents were more likely to report decreasing their purchase of fresh produce compared with food-secure respondents. 

Participants who reported purchasing more or less fresh fruits and vegetables were then prompted with an open-ended question for them to explain why their purchasing habits had changed. The most prevalent themes for each question are found in Table 4. Out of the 151 open-ended responses for those who reported purchasing more fresh fruits and vegetables, the most prevalent reason cited was spending more time at home (*n* = 71). This change in purchasing habits was commonly attributed to children being home more, as well as cooking more meals at home. The second most prevalent reason was the desire to eat healthy (*n* = 58), with many specifically stating they wanted to improve their immune system. In addition, many participants wanted healthy foods available for their children and the rest of the family. Participants (*n* = 27) reported good availability of fresh fruits and vegetables, which was often compared to the poor availability of other foods such as meat, shelf-stable foods, and frozen or canned vegetables. Some participants (*n* = 11) cited the low cost of fresh fruits and vegetables as a reason to purchase more of them, especially in comparison to other foods that had increased in price, such as meat. Lastly, some participants (*n* = 9) reported that they purchased more fresh fruits and vegetables because they or their children like the taste. 

The number of open-ended responses for those who reported purchasing less fresh fruits and vegetables was 134. The most commonly cited reason was poor availability (*n* = 58), which included reports of low supply or poor selection of fresh produce at stores. Many participants (*n* = 29) reported the high price of fresh produce as a reason for purchasing less, stating that the cost of fresh fruits and vegetables had increased or that they had less money to spend on food. Reducing the number of trips to buy was also a common (*n* = 28) reason for purchasing less fresh fruits and vegetables. Participants stated that they go bad quickly in comparison to shelf-stable foods that tend to last longer between trips to the store. Some participants (*n* = 19) reported poor quality of fresh produce, such as being overripe or less fresh. Lastly, some participants reported purchasing less fresh fruits and vegetables due to concerns about coronavirus contamination (*n* = 15). Participants were concerned that fresh produce was touched by other people, and did not want to eat uncooked produce because they were not confident that washing fresh produce would decontaminate it.

## 4. Discussion

This study examined the relationship between food security and fruit and vegetable purchasing and consumption during the COVID-19 pandemic in the U.S. state of Michigan. To date, there is limited published research examining the relationship between food security and fruit and vegetable purchasing or consumption during the COVID-19 pandemic. This research aides in understanding how pandemics and economic crises may impact healthy food consumption and food security. This knowledge is critical for developing strategies to improve diet quality and food security during times of crisis. 

Food insecurity was prevalent among respondents, with 36.2% of them experiencing food insecurity in the last month. It is estimated that early in the COVID-19 pandemic, the U.S. food insecurity rate doubled to 22.8%, primarily driven by the job disruptions caused by state lockdowns [6]. Other studies in the U.S. have also consistently found increases in food insecurity. For example, in a study in the U.S. state of Vermont, 24.8% of the respondents were food-insecure shortly after the beginning of the pandemic, compared with 18.8% before the pandemic [35]. There is also evidence that very low food security, the most severe form of food insecurity, has increased sharply: a study of U.S. families with children found the prevalence of very low food security rose from 10% before the pandemic to 30% during the pandemic [36]. Several U.S. governmental efforts to reduce food insecurity have been enacted since the beginning of the pandemic, most notably changes to the U.S.’s hallmark food assistance program, the Supplementation Nutrition Assistance Program (SNAP). The Families First Coronavirus Act (FFCA) included a number of provisions to boost SNAP benefits: it allowed for increasing benefits to the maximum amount, suspending work requirements, and initiating the Pandemic Electronic Benefits Transfer (P-EBT program). The P-EBT program provided additional SNAP benefits for eligible children during school closures [37]. Subsequent laws have been passed to increase the maximum amount of SNAP benefits. In October 2020, maximum SNAP allotments increased by 5.3% [38], which was later upped to a temporary 15% increase lasting until June 2021 [39]. Additional measures beyond SNAP have been implemented to address the growing food insecurity. Food distribution through food pantries hit record numbers, while simultaneously adapting to mobile formats to reduce COVID-19 exposure risk [40]. Other programs, including pick-up meals for children who are out of school, have been developed or expanded to help address the increased need [41].

In the present study, we found that food-insecure respondents consumed fruits and vegetables fewer times per day than food-secure respondents, and they were more likely to perceive cost as a barrier to eating fruits and vegetables. This is consistent with previous research on the relationship between food insecurity, income, and fruit and vegetable intake [12,14,42]. Food-insecure respondents were also more likely to report reducing their total fruit and vegetable consumption since the pandemic’s start. This difference in fruit and vegetable intake between food-secure and food-insecure individuals is of particular concern during the COVID-19 crisis. Not only does eating adequate amounts of fruits and vegetable benefit the immune system, but also it is associated with reduced risk of chronic diseases [15,16,17,18,19,20]. It is predicted that the pandemic’s effects on dietary intake may have negative long-term health consequences, especially for food-insecure individuals [43]. Several studies have examined changes in fruit and vegetable consumption since the beginning of the COVID-19 pandemic. In a nationwide study of U.S. adults, 28.2% reported consuming less non-starchy vegetables, and 33.4% reported decreased consumption of fruit [44]. Two studies in Italy focused specifically on change in fresh fruit and vegetable intake: in one study, 18% of the respondents reported consuming less fresh vegetables, and about 17% reported consuming less fresh fruit, while in the other study 8.7% of the respondents reported consuming less fresh fruits and vegetables [45,46]. In a study that included respondents from four Western countries, the average portions of fruits and vegetables consumed per day actually increased from before the pandemic to during the pandemic [47]. No studies to date have compared changes in fruit and vegetable intake between food-secure and food-insecure individuals, but one study of U.S. emerging adults found that food-insecure respondents had significantly lower home availability of fruits and vegetables compared with food-secure respondents [48]. 

We also examined if changes in consumption of particular forms of fruits and vegetables differed by food security status. We found that food-insecure respondents were more likely to report decreasing their frozen, fresh, and canned fruit and vegetable consumption compared to food-secure respondents. However, they were also more likely to report increasing their canned fruit and vegetable consumption compared with food-secure respondents. These findings are consistent with other studies that have found increases in the consumption of shelf-stable foods and decreases in that of fresh foods since the beginning of the COVID-19 pandemic [35,36,44,45,46,47]. Several studies have also found that a higher proportion of food-insecure individuals report buying less fresh foods [35,36]. Taken together, this suggests that promoting the consumption of canned fruits and vegetables for food-insecure individuals may be a viable strategy to boost fruit and vegetable intake during pandemics. Since the frequency of grocery shopping has decreased since the pandemic’s start, canned foods have the benefit of low perishability. Canned fruits and vegetables are typically less expensive than both fresh and frozen produce, and frequently have similar nutritional value, allowing for food-insecure households to meet dietary fruit and vegetable recommendations in a more cost-effective way [49,50,51]. Since canned vegetables can contain high levels of sodium and canned fruits can contain added sugar, no-salt-added canned vegetables and canned fruit packed in juice should be recommended [49,50].

For participants who reported purchasing less fresh fruits and vegetables since the pandemic’s outbreak, the most commonly cited reasons were poor availability, high price, reduced store trips, poor quality, and concerns over contamination. Produce availability and quality may have been impacted by in-store food shortages during the pandemic; however, fresh produce has not been among the items commonly reported to be out of stock at grocery stores [52]. Regarding high prices, U.S. Bureau of Labor Statistics data found that consumer prices of fruits and vegetables had a small increase of 2.5% from March to June of 2020 [53]. Reduction in household income from job disruption was likely another financial factor contributing to the perception of cost as a barrier, since low-income households spend less money on fruits and vegetables [42]. Reducing the frequency of grocery store trips to avoid COVID-19 exposure was also a commonly reported reason to reduce fresh produce purchase due to its high perishability. Alternatively, frozen and canned fruits and vegetables are nutritionally similar to fresh produce, but without the problem of high perishability [49,50,51]. Lastly, some participants bought less fresh produce due to concerns over contamination with coronavirus. Other studies in the U.S and beyond have also found concern over food contamination to be common [35,54]. Based on what is known to date, there is no evidence that COVID-19 can be spread from food [55]. For participants who reported purchasing more fresh fruits and vegetables since the pandemic started, common reasons included spending more time at home, a desire to eat more healthily, good availability, low price, and enjoying the taste. The good availability and low price cited by these respondents is in contrast to the high price and poor availability reported by other respondents. This may be indicative of variation among store types or geographies in how COVID-19 affected fresh produce. 

## 5. Conclusions

We found a high prevalence of food insecurity in this study, indicating that adequate food assistance is now more important than ever. There will likely be an increased need for federal and emergency food assistance for a considerable amount of time: the rise in unemployment that has accompanied the pandemic will likely take several years to return to pre-pandemic levels [10]. We also found that food-insecure respondents consumed fruits and vegetables less frequently than food-secure respondents. One of the key strengths of this study is that it compared fruit and vegetable intake between food-secure and food-insecure individuals. To date, other studies examining diet and food security during the COVID-19 pandemic have not examined this. Additionally, this study is unique in that it examined change in intake of different forms of fruits and vegetables (fresh, canned, and frozen) during the COVID-19 pandemic. We found that compared with food-secure respondents, food-insecure respondents were more likely to report consuming less total, fresh, and frozen vegetables then they did before the pandemic. They were also more likely to report consuming more or less canned fruits and vegetables compared with before the pandemic. Food shopping patterns have changed drastically since the pandemic started; thus, it is important to determine which forms of fruits and vegetables are best suited to these altered shopping patterns. Public health messaging about the importance of eating a healthy diet, such as eating fruits and vegetables, during the COVID-19 pandemic is needed. Additionally, this messaging should emphasize that all forms of fruits and vegetables (canned, fresh, and frozen) are nutritious foods. One limitation of this study is that we were unable to calculate the response rate, thus the extent of non-response bias is unknown. Another limitation of this study is the convenience sample design. Both non-response bias and using a convenience sample resulted in an overwhelmingly female sample and resulted in under-representation of racial and ethnic minority respondents. Both before and during the pandemic, food insecurity has been reported to be much more prevalent among Native American, African American, and Hispanic populations, and these populations have also been disproportionately burdened by severe COVID-19 infection and death [56,57]. Additional research is needed to examine dietary habits within marginalized groups during the COVID-19 pandemic and address diet and food insecurity disparities.

## Figures and Tables

**Table 1:** Demographic characteristics of respondents (*n* = 484).

	Food-Secure Number(%) ^a^	Food- Insecure Number(%) ^a^	Total Number(%) ^a^	*p*-Value
Age: Mean ± SD	46.8 ± 12.6	44.7 ± 11.3	46.0 ± 12.2	0.053
Race				0.069
American Indian or Alaska Native	2 (0.7)	0 (0.0)	2 (0.4)	
Asian	2 (0.7)	1 (0.6)	3 (0.6)	
Black or African American	10 (3.3)	16 (9.1)	26 (5.4)	
Native Hawaiian or Other Pacific Islander	0 (0.0)	1 (0.6)	1 (0.2)	
White	282 (91.9)	150 (85.7)	432 (89.6)	
Other Race	7 (2.3)	4 (2.3)	11 (2.3)	
Multiple Races	4 (1.3)	3 (1.7)	7 (1.5)	
Ethnicity				0.042 *
Not Hispanic or Latino/a	302 (97.7)	164 (93.7)	466 (96.3)	
Hispanic or Latino/a	7 (2.3)	11 (6.3)	18 (3.7)	
Gender				0.016 *
Male	13 (4.2)	1 (0.6)	14 (2.9)	
Female	293 (94.8)	169 (96.6)	462 (95.5)	
Other	3 (1.0)	5 (2.9)	8 (1.7)	
Child in household				<0.001 *
No	174 (57.4)	66 (37.9)	240 (50.3)	
Yes	129 (42.6)	108 (62.1)	237 (49.7)	
Education				<0.001 *
High school/GED or less	17 (5.5)	36 (20.6)	53 (11.0)	
Some college	56 (18.1)	61 (34.9)	117 (24.2)	
Associates/tech school/ apprenticeship	45 (14.6)	37 (21.1)	82 (16.9)	
Bachelor’s degree	97 (31.4)	24 (13.7)	121 (25.0)	
Graduate/professional degree	94 (30.4)	17 (9.7)	111 (22.9)	
Income				<0.001 *
<$24,999	45 (14.8)	70 (40.1)	115 (24.2)	
$25,000 to $49,999	73 (24.2)	57 (32.7)	130 (27.4)	
$50,000 to $99,999	114 (37.7)	40 (23.0)	154 (32.4)	
$100,000 or more	70 (23.2)	7 (4.1)	77 (16.1)	
Employment disruption				<0.001 *
Yes	159 (53.9)	120 (75.5)	279 (61.5)	
No	136 (46.1)	39 (24.5)	175 (38.6)	

^a^ Column percentage, * indicates significant differences between food-secure and food-insecure respondents at the *p* < 0.05 level.

**Table 2:** Fruit and vegetable outcome variable response frequency by food security status.

	Food-Secure Number (%)	Food-Insecure Number (%)
Times per day consumed fruit and vegetables (mean ±SD)	3.33 ± 1.98	2.99 ± 2.35
Change in canned fruit and vegetable consumption		
Less	34 (11.2)	59 (34.1)
The same	216 (70.8)	61 (35.3)
More	55 (18.0)	53 (30.6)
Change in frozen fruit and vegetable consumption		
Less	38 (12.4)	56 (32.2)
The same	200 (65.4)	70 (40.2)
More	68 (22.2)	48 (27.6)
Change in fresh fruit and vegetable consumption		
Less	71 (23.1)	80 (46.0)
The same	115 (37.5)	46 (26.4)
More	121 (39.4)	48 (27.6)
Change in total fruit and vegetable consumption		
Less	47 (15.4)	76 (43.4)
The same	164 (53.6)	61 (34.9)
More	95 (31.1)	38 (21.7)
Change in fresh fruit and vegetable purchase		
A lot less	15 (4.9)	20 (11.6)
A little less	48 (15.6)	48 (27.8)
The same	138 (44.8)	59 (34.1)
A little more	77 (25.0)	29 (16.8)
A lot more	30 (9.7)	17 (9.8)
Cost is a barrier to eating fruits and vegetables		
Strongly disagree	97 (31.5)	18 (10.3)
Somewhat disagree	64 (20.8)	10 (5.8)
Neither agree nor disagree	60 (19.5)	32 (18.4)
Somewhat agree	73 (23.7)	64 (36.8)
Strongly agree	14 (4.6)	50 (28.7)
Produce in neighborhood is high-quality		
Strongly disagree	23 (7.5)	12 (6.9)
Somewhat disagree	37 (12.0)	36 (20.6)
Neither agree nor disagree	41 (13.3)	50 (28.6)
Somewhat agree	114 (37.0)	61 (34.9)
Strongly agree	93 (30.2)	16 (9.1)

**Table 3:** Generalized ordered logistic regression models: change in fruit and vegetable consumption, purchase, and perceived barriers in food-insecure vs. food-secure respondents.

	Model 1 ^a^			Model 2 ^b^		
	OR(SE) ^c^	(95% CI) ^d^	*p*-Value	OR (SE)	(95% CI)	*p*-Value
Change in fresh fruit and vegetable consumption						
Less	0.35 (0.07)	(0.24, 0.53)	<0.001 *	0.33 (0.07)	(0.22, 0.50)	<0.001 *
Same	0.59 (0.12)	(0.39, 0.88)	0.009 *	0.53 (0.11)	(0.35, 0.81)	0.003 *
Change in frozen fruit and vegetable consumption						
Less	0.30 (0.07)	(0.19, 0.48)	<0.001 *	0.28 (0.07)	(0.18, 0.46)	<0.001 *
Same	1.33 (0.29)	(0.87, 2.05)	0.188	1.21 (0.27)	(0.78, 1.89)	0.391
Change in canned fruit and vegetable consumption						
Less	0.24 (0.06)	(0.15, 0.39)	<0.001 *	0.22 (0.06)	(0.13, 0.36)	<0.001 *
Same	2.01 (0.45)	(1.30, 3.10)	0.002 *	1.95 (0.45)	(1.24, 3.05)	0.004 *
Change in total fruit and vegetable consumption						
Less	0.24 (0.05)	(0.15, 0.36)	<0.001 *	0.22 (0.05)	(0.14, 0.34)	<0.001 *
Same	0.62 (0.14)	(0.40, 0.95)	0.028 *	0.56 (0.13)	(0.36, 0.88)	0.012 *
Change in fresh fruit and vegetable purchase						
A lot less	0.39 (0.14)	(0.19, 0.79)	0.008 *	0.34 (0.13)	(0.17, 0.71)	0.004 *
A little less	0.40 (0.08)	(0.26, 0.60)	<0.001 *	0.39 (0.08)	(0.26, 0.59)	<0.001 *
Same	0.68 (0.14)	(0.45, 1.03)	0.066	0.62 (0.13)	(0.40, 0.94)	0.023 *
A little more	1.01 (0.32)	(0.54, 1.89)	0.976	0.91 (0.30)	(0.48, 1.73)	0.782
Cost is a barrier to eating fruits and vegetables						
Strongly disagree	3.98 (1.11)	(2.31, 6.86)	<0.001 *	3.86 (1.09)	(2.22, 6.71)	<0.001 *
Somewhat disagree	5.71 (1.35)	(3.60, 9.07)	<0.001 *	5.84 (1.41)	(3.64, 9.38)	<0.001 *
Neither agree nor disagree	4.83 (0.98)	(3.24, 7.19)	<0.001 *	4.78 (0.99)	(3.17, 7.18)	<0.001 *
Somewhat agree	8.47 (2.72)	(4.52, 15.88)	<0.001 *	7.95 (2.57)	(4.22, 14.97)	<0.001 *
Produce in neighborhood is high-quality						
Strongly disagree	1.10 (0.40)	(0.53, 2.26)	0.804	1.22 (0.46)	(0.59, 2.53)	0.594
Somewhat disagree	0.64 (0.14)	(0.41, 0.99)	0.045 *	0.69 (0.16)	(0.44, 1.07)	0.099
Neither agree nor disagree	0.38 (0.07)	(0.26, 0.56)	<0.001 *	0.41 (0.08)	(0.28, 0.61)	<0.001 *
Somewhat agree	0.23 (0.07)	(0.13, 0.41)	<0.001 *	0.25 (0.07)	(0.14, 0.44)	<0.001 *

^a^ Model 1 is unadjusted, ^b^ Model 2 is adjusted for age and race/ethnicity, ^c^ OR = odds ratio, SE = standard error. Odds ratios are presented as the ratio of the odds of food-insecure to food-secure respondents (OR < 1 indicates reduced odds for food-insecure respondents, OR >1 indicates increased odds for food-insecure respondents), ^d^ CI = confidence interval, * indicates significant difference by food security status at the *p* < 0.05 level.

**Table 4:** Qualitative themes–reasons for purchasing more or less fresh fruits and vegetables.

Question	Themes	Example Quotes
Please explain why you are purchasing more fresh fruits and vegetables (*n* = 151)	More Time at Home (*n* = 71)	“Kids are home and not packing or buying a lunch at school. This means we have to have lunch options at home due to more family members at home.”
	Healthy (*n* = 58)	“I’ve started caring more about my family’s overall health since the pandemic started. Switching to a healthier diet with more fruits and veggies.”
	Good Availability (*n* = 27)	“At times the only place that I knew there would be food was in the fresh food aisles, so I planned more fresh dinners.”
	Low Price (*n* = 11)	“Produce is one thing that has been available reliably and with less cost impact, so I am making more meals with produce.”
	Taste (*n* = 9)	“My children like them. They may be hard to find, I like to stock up now so don’t have to go out much.”
Please explain why you are purchasing less fresh fruits and vegetables (*n* = 134)	Poor Availability (*n* = 58)	“There haven’t been enough to purchase. With the low supply of most items, I did not want to take away from another family.”
	High Price (*n* = 29)	“Prices are too high for me. We have much less to spend on food and prices have gone up.”
	Reduced Store Trips (*n* = 28)	“With trips to the store being further apart to avoid exposure, fruits and veggies don’t keep well. When you don’t go to the store every week, frozen veggies are the only good choice.”
	Poor Quality (*n* = 19)	“The store doesn’t have good produce quality and little to no variety. I sometimes struggle to find tomatoes and green peppers.”
	Contamination (*n* = 15)	“I do not trust that they are safe to eat. Many people walk by them and breathe on them throughout the day, and that worries me.”

## Data Availability

The data are not publicly available due to participant confidentiality.
